# The Interaction Between Insulin Resistance and Neuroinflammation in the Brain and Its Impact on Diabetic Encephalopathy

**DOI:** 10.3390/biology15130990

**Published:** 2026-06-23

**Authors:** Yiheng Wang, Hanyu Li, Xiaoxu Yang, Mei Yang, Wei Hu, Xihua Cheng, Yancheng Zhong

**Affiliations:** 1 Hunan Key Laboratory of Vascular Biology and Translational Medicine, Medical School, Hunan University of Chinese Medicine, Changsha 410208, China; wangyiheng@stu.hnucm.edu.cn (Y.W.); 202310010111@stu.hnucm.edu.cn (H.L.); 004367@hnucm.edu.cn (X.Y.); 003779@hnucm.edu.cn (M.Y.); 2 Department of Integrated Traditional Chinese and Western Medicine, Xiangya Boai Rehabilitation Hospital, Changsha 410151, China; huwei5_2001@126.com

**Keywords:** brain insulin resistance, neuroinflammation, diabetic encephalopathy, interplay, treatment

## Abstract

Diabetic encephalopathy (DE) is a severe complication of diabetes mellitus affecting the central nervous system (CNS), characterized by cognitive dysfunction. This review systematically explores how the interplay between brain insulin resistance (BIR) and neuroinflammation contributes to the pathogenesis of DE. BIR refers to the diminished responsiveness of the CNS to insulin signaling, resulting in the suppression of the PI3K/Akt and MAPK pathways, impaired glucose metabolism, and dysfunction across multiple neural cell types—including neurons, astrocytes, microglia, brain endothelial cells, and oligodendrocytes. These disturbances manifest as impaired energy metabolism, compromised synaptic plasticity, disruption of the blood–brain barrier, and reduced myelination. Importantly, BIR and neuroinflammation form a vicious cycle within these cells, mutually exacerbating each other and jointly driving the pathological progression of DE. Finally, we have compiled a list of currently available drugs that can improve BIR and suppress neuroinflammation, along with the latest progress in clinical trials, to provide new insights for the future of precision treatment for DE.

## Simple Summary

Diabetes actively impacts the central nervous system, leading to a condition termed diabetic encephalopathy that results in cognitive decline and memory loss. Brain insulin resistance and neuroinflammation represent the two primary pathological mechanisms driving this cognitive impairment. This review details how these two factors interact through a reciprocal feedback loop, where diminished insulin signaling triggers inflammatory responses, and elevated inflammation conversely exacerbates insulin resistance. This intertwined process ultimately disrupts brain energy metabolism, damages diverse neural cell networks, impairs synaptic connections, and compromises the blood–-brain barrier. By clarifying these interactive mechanisms, this paper highlights therapeutic approaches that concurrently target insulin resistance and inflammation to prevent cognitive decline in diabetic patients.

## 1. Introduction

Brain insulin resistance (BIR) refers to a significant reduction in the central nervous system’s (CNS) ability to respond to insulin signals. Its core manifestation is a decrease in insulin receptor (IR) sensitivity in cognitive and metabolic brain regions such as the hippocampus, hypothalamus, and cortex, which in turn inhibits downstream PI3K/Akt and MAPK signaling pathways [1,2,3,4]. Intracerebral insulin is primarily secreted by peripheral pancreatic β-cells and enters the brain via brain endothelial cells (BECs) across the blood–brain barrier (BBB) [5,6]. However, recent studies have found that neurons [7,8], astrocytes [9], and choroid plexus epithelial cells [10,11,12] also possess the ability to synthesize and secrete these substances on their own. Therefore, the onset of BIR represents a pathological network of multicellular dysregulation involving neurons [13], astrocytes [14], microglia [15], and the cerebral microvascular system [16]. When insulin transport or IR function in the brain is impaired, pathway inhibition triggers a cascade of events leading to impaired glucose transport, mitochondrial damage, neuroinflammation, oxidative stress, apoptosis, and dysregulation of autophagy. When cerebral insulin transport or insulin receptor function is impaired, inhibition of this signaling pathway triggers a cascade of reactions, leading to glucose transport deficits [17], mitochondrial dysfunction [18], neuroinflammation [19], oxidative stress [20], apoptosis [21], and dysregulated autophagy [22], thereby impairing metabolism [23], synaptic plasticity [24], and cognitive memory function [25]. Ultimately, these disturbances drive the development of a range of neurological, psychiatric, and metabolic disorders, including Alzheimer’s disease (AD), Parkinson’s disease (PD), and diabetic encephalopathy (DE).

DE, as a highly detrimental chronic central nervous system complication of diabetes mellitus, is clinically characterized by progressive cognitive decline and learning and memory impairments, and serves as a significant independent risk factor for the subsequent development of AD in diabetic patients [26]. Sustained systemic hyperglycemia and lipid metabolism disorders serve as the pathological cornerstone of DE, directly mediating neurodegeneration in cognition-critical brain regions such as the hippocampus by triggering intense oxidative stress and neuroinflammation [27]. Given the high similarity in clinical cognitive impairment between DE and AD, as well as the profound overlap in downstream pathological pathways such as BIR and inflammatory cascades, some scholars have classified AD as a special manifestation of DE. However, from the perspective of etiological origins, DE has a clear boundary from the classical neurodegenerative diseases represented by AD. The central nervous system damage in AD is primarily anchored to the misfolding and abnormal deposition of specific proteins, such as extracellular β-amyloid (Aβ) and intracellular Tau protein tangles [28]; in contrast, the pathogenesis of DE is a process of core metabolic reprogramming in the brain driven by peripheral hyperglycemia, which triggers aberrant insulin signaling, impaired mitochondrial function, accompanied by oxidative stress, neuroinflammation, and glucolipid metabolic disorders, collectively leading to an energy metabolism crisis, synaptic damage, and accelerated brain aging [29].

This review focuses on the vicious interplay between BIR and neuroinflammation in DE, unlike previous studies on isolated mechanisms. It systematically describes how BIR-induced pathway inhibition and glucose dysregulation cascade with neuroinflammation, and innovatively maps the cellular pathology from a pan-cellular network perspective—covering neuronal, glial, endothelial, and oligodendrocytic dysfunction. It further links these mechanisms to clinical translation, evaluates current therapies, and offers new theoretical and methodological insights to break the DE cascade and enable central precision therapy.

## 2. Mechanism of BIR

### 2.1. BIR in Metabolic Diseases

#### 2.1.1. Obesity

Long-term consumption of a high-fat diet (HFD) is strongly correlated with neuroinflammation and BIR [30]. It has been demonstrated that HFD elevates circulating free fatty acid (FFA) levels, thereby promoting ceramide synthesis [31]. Ceramides can activate the nuclear factor kappa-B (NF-κB) signaling pathway and directly enhance NADPH oxidase (NOX) activity, leading to oxidative stress and insulin resistance [32]. The accumulation of these metabolites in the brain ultimately inhibits the phosphorylation of IR, resulting in BIR. Furthermore, elevated levels of palmitic acid (PA), which have been detected in the cerebrospinal fluid and brain of obese patients as well as in the hypothalamus of mice fed a long-term HFD, can also induce BIR. [33]. On one hand, PA inhibits the phosphorylation of IR tyrosine residues [34], directly causing and aggravating BIR. On the other hand, it promotes the membrane translocation of PKC-θ, increasing the phosphorylation of insulin receptor substrate 1 (IRS-1) at Ser307, thereby suppressing the activation of the insulin/PI3K/Akt pathway [35]. Hypothalamic inflammation is considered a primary mechanism in obesity-induced BIR. Inflammatory changes are detectable in the brains of HFD-fed animals, and researchers have observed increased ROS production and upregulated NF-κB signaling in the hypothalamus of obese animals [36]. The activation of NF-κB signaling induces the expression of suppressor of cytokine signaling 3 (SOCS3) in the hypothalamus. SOCS3 promotes the proteasomal degradation of IRS-1/2, thereby impairing insulin-dependent phosphorylation of IR and its downstream signaling [37,38]. Furthermore, obesity-induced overactivation of macrophages stimulates the robust production and release of tumor necrosis factor-α (TNF-α) and interleukin-6 (IL-6) [39]. These pro-inflammatory cytokines not only directly impair insulin signaling but also activate c-Jun N-terminal kinase (JNK). JNK promotes the serine phosphorylation of IRS-1, which inhibits its tyrosine phosphorylation and downstream signaling, ultimately leading to hypothalamic insulin resistance [40].

#### 2.1.2. Type 2 Diabetes Mellitus

Type 2 diabetes mellitus (T2DM) often presents with hyperinsulinemia. Hyperinsulinemia downregulates the protein levels of IR in BECs at the BBB through a negative feedback mechanism. A study conducted on human cerebral microvascular endothelial cells (hCMEC/D3) found that when cells were exposed to insulin for a short period (15 min), the IR signaling pathway was activated. However, when the exposure was prolonged to 300 min (5 h), the protein expression level of the IR-β subunit showed a significant and substantial decrease [41]. Concurrently, sustained high levels of insulin continuously activate and accelerate the internalization process of IR, ultimately reducing the number of receptors on the cell membrane and hindering insulin’s entry into the cell [42,43]. Furthermore, hyperinsulinemia increases serine/threonine phosphorylation of IRS, which blocks the downstream PI3K-Akt pathway of insulin signaling [44].

A hyperglycemic environment enhances the paracellular permeability of the BBB, which is closely associated with reduced expression of the tight junction proteins occludin and zonula occludens-1 (ZO-1) in endothelial cells [45]. This disruption of BBB integrity facilitates the entry of increased amounts of resistin into the brain. Resistin that enters the brain exacerbates metabolic dysregulation through multiple mechanisms. Firstly, it directly blocks the insulin signaling transduction pathway by inhibiting the activity of IR and its downstream signaling molecule Akt. Furthermore, recent studies have revealed a novel pathway through which resistin induces hypothalamic inflammation: resistin binds to Toll-like receptor 4 (TLR4) on the surface of neurons or glial cells in the hypothalamus and brainstem, thereby activating key inflammatory signaling pathways such as NF-κB, JNK, and p38 MAPK [46]. This cascade of activation not only directly triggers a central inflammatory response but also further disrupts glucose homeostasis, creating a vicious cycle. Furthermore, in T2DM mouse models and insulin-resistant cell models, sustained hyperglycemia and hyperlipidemia induce neuronal insulin resistance via downregulation of the PI3K/Akt pathway, activate glycogen synthase kinase 3β (GSK-3β) to promote microtubule-associated protein tau hyperphosphorylation, and promote the aberrant expression of amyloid precursor protein (APP), ultimately leading to cognitive dysfunction in mice [47,48].

Leptin is a hormone secreted by adipocytes, primarily responsible for regulating energy balance and appetite. However, in patients with obesity and T2DM, leptin levels are often significantly elevated and accompanied by resistance [49]. Studies indicate that the PI3K pathway serves as a central node in both insulin and leptin signaling, playing a synergistic role, particularly in the regulation of energy metabolism. Prolonged exposure to high levels of leptin enhances the binding capacity of SOCS3 to the IR, thereby impairing brain insulin sensitivity [50]. Specifically, SOCS3 blocks insulin signal transduction by interfering with the interaction between IR and IRS-2. Additionally, leptin itself exhibits pro-inflammatory effects. In T2DM, leptin resistance may lead to increased secretion of inflammatory factors such as TNF-α and IL-6 from adipose tissue [51]. Upon entering the brain, these inflammatory cytokines can inhibit the tyrosine phosphorylation of IRS-1, ultimately contributing to BIR [52].

**Figure 1:**
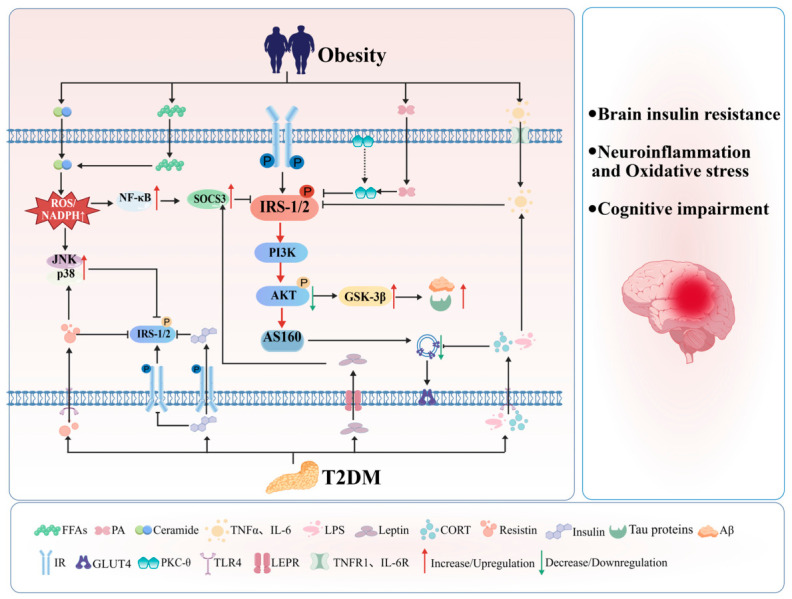
The mechanisms underlying the development of BIR in metabolic diseases. Obesity and T2DM induce BIR through multiple intertwined pathways. In the context of obesity, HFD leads to the accumulation of circulating FFAs and PA in the brain. FFAs promote ceramide synthesis and activate NF-κB, enhancing NOX activity and triggering oxidative stress. Ceramides and PA directly inhibit IR phosphorylation; PA also promotes IRS-1 Ser307 phosphorylation via PKC-θ translocation, thereby blocking the PI3K/Akt pathway. Furthermore, NF-κB activation upregulates SOCS3, which mediates the degradation of IRS-1/2. Peripheral pro-inflammatory cytokines (TNF-α, IL-6) enter the brain and activate JNK, exacerbating serine phosphorylation of IRS-1. In the context of T2DM pathology, hyperinsulinemia accelerates the internalization and degradation of IR at the BBB and inhibits the PI3K/Akt pathway. Hyperglycemia disrupts tight junction proteins (Occludin/ZO-1), leading to increased BBB permeability and facilitating resistin entry into the brain. Resistin not only inhibits IR/Akt signaling but also activates NF-κB, JNK, and p38 MAPK via TLR4, triggering inflammation. Concurrently, high glucose and high lipids activate GSK-3β, promoting Tau hyperphosphorylation. Additionally, leptin resistance enhances the binding of SOCS3 to IR, blocking the IR/IRS-2 interaction. Finally, gut–brain axis (GBA) dysregulation mediates LPS endotoxemia and activates the HPA axis, resulting in elevated corticosterone levels, which comprehensively inhibit IR phosphorylation and GLUT4 membrane translocation in the hippocampus, ultimately exacerbating neuroinflammation, oxidative stress, BIR, and cognitive impairment. Created with BioGDP.com [59].

Accumulating evidence indicates that dysregulation of the gut–brain axis (GBA) is an important mechanism promoting the development of BIR, primarily mediated through the dual pathways of immune inflammation and neuroendocrine signaling [53]. On one hand, gut microbiota dysbiosis induced by long-term hyperglycemia disrupts the integrity of the intestinal barrier [54], leading to the entry of Gram-negative bacteria-derived lipopolysaccharides (LPS) into the bloodstream, thereby inducing metabolic endotoxemia [55,56]; the chronic low-grade systemic inflammation triggered by LPS not only interferes with peripheral insulin signal transduction but also affects CNS via the GBA [57]. On the other hand, the gut microbiota and its metabolites can directly communicate with the brain via vagal sensory pathways and the local enteric nervous system (ENS), and abnormally activate the hypothalamic–pituitary–adrenal (HPA) axis [54]. Patients with T2DM typically exhibit abnormal activation of the HPA axis, leading to a significant elevation in circulating corticosterone (CORT) levels [56]. Elevated levels of CORT impair central insulin signal transduction in the rat hippocampus. In the hippocampus of CORT-treated rats, insulin-stimulated IR phosphorylation, total Akt content, and total glucose transporter 4 (GLUT4) content were all reduced, and insulin-stimulated translocation of GLUT4 to the plasma membrane in the hippocampus was completely abolished [58]. The specific mechanisms underlying the development of BIR in metabolic diseases are illustrated in Figure 1.

### 2.2. BIR Induced by Intracerebral Cellular Abnormalities

#### 2.2.1. Reduced Secretion of Endogenous Insulin by Brain Cells

The origin of insulin within the CNS has long been debated. The traditional view holds that insulin in the brain is primarily derived from peripheral pancreatic secretion and enters the brain by crossing the BBB. However, recent studies have confirmed the existence of endogenous de novo synthesis of insulin in the brain. Studies have shown that approximately 3–5% of rabbit brain neurons express insulin mRNA and protein [60], and the rat brain as well as the periventricular nucleus of the hypothalamus also clearly express the insulin II gene (Ins2) [3,61]. In addition to neurons, glial cells are also involved in local insulin supply under specific conditions. For example, certain GABAergic glial cells can synthesize and release insulin in response to changes in glucose concentration [62]; primary cultured astrocytes can also autonomously express preproinsulin mRNA and protein [9].

However, under pathological conditions such as T2DM, AD, or neuroinflammation, due to the obstruction of the Wnt3/NeuroD1 pathway or the dramatic elevation of intracellular ROS and mitochondrial oxidative stress damage induced by Aβ and LPS, endogenous insulin synthesis in neurons and astrocytes becomes severely insufficient [63]. This insulin deficiency disrupts neural network homeostasis, leading to insufficient IR phosphorylation and consequently preventing the effective activation of downstream local PI3K/Akt or MAPK pathways. As a result, insulin-like growth factor 1 (IGF-1), which acts synergistically with insulin, is also unable to effectively activate its receptor. Under normal physiological conditions, activated astrocytes secrete IGF-1 and activate the PI3K/Akt pathway in adjacent cells via paracrine signaling, thereby promoting neuronal proliferation and enhancing the expression of the glutamate transporter GLT-1. However, when this mechanism is inactivated due to insulin deficiency, the homeostasis of neurotransmitters in the synaptic cleft becomes severely disrupted [64]. Furthermore, insufficient autocrine insulin secretion from neurons also prevents the PI3K/Akt pathway from promoting GLUT4 translocation to the cell membrane, thereby significantly reducing glucose uptake efficiency in brain regions during activities such as learning and memory [17,65]. This insufficient endogenous insulin synthesis in the brain, resulting from the dysregulation of specific signaling pathways and oxidative stress, is closely associated with cognitive impairment, decreased synaptic plasticity, and enhanced neuroinflammation.

#### 2.2.2. Insulin Receptor Dysfunction in Brain Cells

The sensitivity of IR is crucial for insulin signal transduction. Studies indicate that the apolipoprotein E4 (APOE4) allele exhibits a significant synergistic effect with IR dysfunction. In an astrocyte-specific IR knockout (GIRKO) model, the excessive secretion of APOE4 interacts with neuronal IR, trapping it in the endosomal compartment [66]. This process not only accelerates internalization, leading to a reduction in cell surface IR density [67,68], but also impedes receptor recycling. Concurrently, this mechanism inhibits mitochondrial complex IV activity and impairs insulin-stimulated mitochondrial respiration and glycolysis, resulting in a collapse of neuronal energy metabolism and creating a vicious cycle of sustained insulin signaling impairment. Notably, the decoupling between mitochondria and the endoplasmic reticulum (ER) leads to overactivation of the unfolded protein response (UPR), where the PERK-pathway-mediated phosphorylation of eIF2α suppresses IR protein synthesis efficiency [69,70].

**Figure 2:**
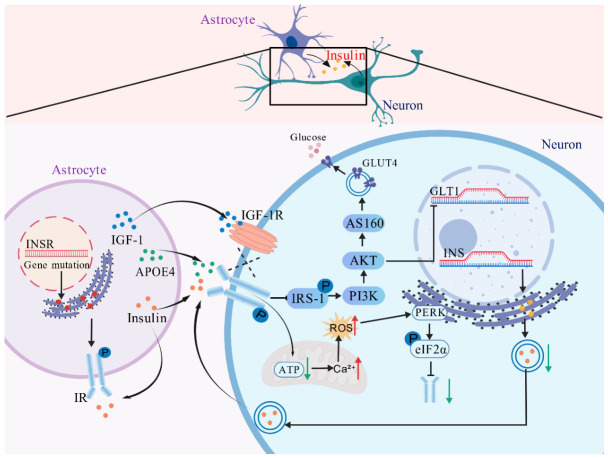
The mechanisms of BIR induced by intracerebral cellular abnormalities. Aberrant insulin signaling in the brain is driven by two mechanisms. The first is insufficient endogenous insulin synthesis: under pathological conditions such as T2DM and AD, the obstruction of the Wnt3/NeuroD1 pathway and ROS-induced mitochondrial oxidative stress reduce insulin secretion from neurons and astrocytes themselves, leading to insufficient IR phosphorylation and ineffective activation of the PI3K/Akt and MAPK pathways. This not only weakens the paracrine protective effect of IGF-1 (e.g., reduced GLT1 expression) but also inhibits GLUT4 membrane translocation, thereby decreasing the glucose uptake efficiency associated with learning and memory. The second mechanism is IR dysfunction: the APOE4 allele reduces membrane receptor density by accelerating IR internalization and impairing its recycling, while also compromising mitochondrial respiration. Mitochondria–endoplasmic reticulum coupling dysfunction inhibits IR synthesis through excessive activation of PERK/eIF2α. Genetic and maternal environmental factors can lead to reduced IR expression and metabolic programming defects in offspring via epigenetic modifications or interference with neural development. Created with BioGDP.com [59].

From a genetic perspective, mutations in the insulin receptor gene (*INSR*) constitute a major mechanism leading to insulin receptor deficiency. The decline in insulin receptor sensitivity in brain cells exhibits a notable pattern of intergenerational inheritance. Studies have found that a maternal high-fat diet can lead to reduced expression of genes such as hypothalamic IR and IRS-2, as well as abnormal leptin signaling, in offspring via DNA methylation modifications, for example, hypomethylation of the SOCS3 promoter in sperm or oocytes [71]. This epigenetic memory can persistently affect the metabolic programming of individuals across multiple generations. Furthermore, alterations in the intrauterine environment during fetal development can also induce BIR in the fetus. Additional research indicates that during critical periods of embryonic development, maternal hyperglycemia or obesity can interfere with normal neuronal differentiation in the fetal brain, reducing neuron numbers and disrupting the formation of insulin-sensitive neuronal circuits, thereby laying the foundation for lifelong impaired metabolic regulation [72,73]. The specific mechanism of BIR induced by intracerebral cellular abnormalities is illustrated in Figure 2.

## 3. Interaction Between BIR and Neuroinflammation

### 3.1. Neuron

BIR triggers mitochondrial dysfunction, directly disrupting neuronal energy homeostasis. BIR induces excessive mitochondrial fission by activating the mitochondrial fission protein DRP1 and downregulating the fusion protein Mfn2, thereby reducing ATP synthesis efficiency and generating oxidative stress [74,75]. Meanwhile, by modulating AMPK activity, BIR affects the localization of Pink1 mRNA and the function of PINK1 on mitochondria, leading to impaired mitophagy and ultimately causing mitochondrial dysfunction [76]. Mitochondrial dysfunction and insufficient ATP synthesis form a vicious cycle, further weakening neuronal energy supply. In addition, while inducing mitochondrial damage, BIR also reduces glucose uptake in the hippocampus, inhibits the GSH-GPX4 antioxidant axis, activates lipid peroxidation, and promotes iron accumulation, thereby triggering neuronal ferroptosis [77]. BIR and mitochondrial dysfunction also promote the onset and progression of DE. In a high-glucose or combined high-glucose and high-lipid environment, activation of the p35/CDK5 pathway on one hand induces insulin resistance in SH-SY5Y cells by inhibiting the PI3K/PKB/mTOR/S6K1 signaling axis, and on the other hand triggers mitochondrial dysfunction and oxidative stress. Together, these events accelerate APP expression, Aβ deposition, and Tau hyperphosphorylation, ultimately leading to cognitive dysfunctions, including impairments in spatial learning and memory, in diabetic mice [78].

Aberration of the PI3K/Akt pathway plays a key role in T2DM-induced neuronal apoptosis. Studies have shown that in the BIR rat model, inhibition of the PI3K/Akt pathway leads to upregulation of the pro-apoptotic protein Bax, downregulation of the anti-apoptotic protein Bcl-2, and activation of Caspase-3, which ultimately results in neuronal damage and apoptosis. Alpha-lipoic acid (ALA), however, can inhibit this process [21].

Impairment of neuronal insulin signaling drives neuroinflammatory cascades. It has been demonstrated that under BIR conditions, the binding between Kelch-like ECH-associated protein 1 (KEAP1) and IκB kinase (IKK) is enhanced, thereby activating the IKK/NF-κB pathway and promoting the release of pro-inflammatory factors such as TNF-α and IL-6. Meanwhile, the interaction between NF-κB and factors including XBP1 and Nrf2 is strengthened, amplifying both inflammatory and oxidative stress responses [79]. Studies have also shown that in the hippocampal tissues of db/db mice, activation of the NLRP3 inflammasome is significantly increased, accompanied by elevated activity of caspase-1 and increased release of the pro-inflammatory cytokines IL-1β and IL-18, thereby exacerbating neuroinflammation [80,81].

### 3.2. Astrocyte

BIR promotes the transformation of astrocytes into the harmful neurotoxic A1 phenotype via neuroinflammation. In diabetic mouse models induced by GRX2 deficiency [82] or intracerebroventricular injection of streptozotocin (STZ) [83], upregulated expression of pro-inflammatory factors IL-1β and TNF-α in hippocampal tissue significantly increases reactive astrocyte markers (such as GFAP and COX2), driving the transition to the A1 phenotype. In addition, activated microglia release pro-inflammatory factors, including TNF-α and IL-6, excessively activating the NF-κB pathway, which can also directly trigger A1 phenotype transformation [84,85]. Notably, NF-κB signaling itself can induce BIR [86], thereby forming a vicious cycle between neuroinflammation and BIR.

Under normal conditions, insulin or IGF-1 inhibits the transcription of autophagy-related genes (such as *p62* and *ULK1/2*) via IR and IGF1R to maintain protein homeostasis [22]. However, in the absence of IR, although autophagy-related proteins are upregulated at the transcriptional level, mitochondrial dysfunction and lysosomal impairment still occur [14]. Studies have found that in astrocytes lacking IR, the loss of IR leads to reduced ATP production in mitochondria and the accumulation of ROS, forming a vicious cycle. This metabolic defect directly inhibits the fusion of autophagosomes with lysosomes, resulting in a decreased LC3-II/LC3-I ratio and accumulation of the autophagy substrate p62, ultimately manifesting as impaired autophagy [87]. This mechanism may represent a key pathological basis for diabetes-related neurodegenerative diseases.

BIR disrupts the PI3K/Akt signaling axis in astrocytes, impairing glutamate cycling and leading to excitotoxicity. Previous studies have indicated that IGF-1 shares the PI3K/Akt signaling pathway with insulin [22]. In astrocytes, loss of the IGF-1 receptor (IGF1R) reduces the membrane localization of the glutamate transporter GLT-1 and downregulates the activity of glutamine synthetase (GS), resulting in decreased rates of glutamate uptake and conversion to glutamine [88]. This preferentially creates an excitotoxic environment. Research also shows that BIR downregulates the astrocytic glutamate transporters GLT-1 and glutamate–aspartate transporter (GLAST), leading to excessive accumulation of glutamate in the synaptic cleft [89]. This causes neuronal hyperexcitation and may ultimately result in neuronal damage or death.

### 3.3. Microglia

Although the broader impact of impaired insulin signaling on microglial activation remains incompletely understood, existing evidence indicates that both microglia and astrocytes can undergo active gliosis and thereby promote inflammatory responses after insulin resistance is induced in the brain by STZ [90]. Researchers have observed microglial activation and upregulated expression of pro-inflammatory factors in various brain regions of HFD-fed mice [91,92]. Minocycline has been shown to reduce hypothalamic microglial activation and improve metabolic dysfunction in HFD-induced mice [93]. Studies have found that levels of HMGB1, TLR4, and NF-κB are significantly increased in the hippocampus of diabetic mice, along with elevated TNF-α, IL-1β, and IL-6 [94]. This suggests that BIR may lead to activation of the HMGB1/TLR4/NF-κB pathway, thereby exacerbating hippocampal inflammatory damage. Meanwhile, HFD can also activate the PERK pathway, triggering the unfolded protein response (UPR). This stress state further activates inflammatory signaling pathways such as NF-κB and JNK, inducing the expression of pro-inflammatory cytokines (e.g., IL-6, TNF-α) and promoting the expression of apoptosis-related proteins (e.g., caspase-3, CHOP) [95]. Other studies indicate that BIR induced by short-term HFD can rapidly trigger hypothalamic microglial inflammation by activating the cGAS/STING innate immune pathway. Recent studies have revealed that GBA imbalance can influence central immune homeostasis by modulating the composition of the gut microbiota and its metabolites, such as short-chain fatty acids and bile acids. Gut microbiota dysbiosis leads to increased pro-inflammatory signals, which are transmitted to the brain via the circulatory or neuroendocrine pathways, inducing the activation of microglia and astrocytes. These activated glial cells further release inflammatory mediators, including TNF-α, IL-1β, and IL-6, thereby triggering neuroinflammation, synaptic dysfunction, and cognitive impairment [96,97,98].

The occurrence of microglial inflammation under BIR conditions also exhibits age and sex differences. In in vivo experiments, insulin continuously activated microglia and increased the expression of COX-2/IL-1β in the hippocampus of young male rats, whereas this effect was not observed in aged rats [99]. This indicates that excessive insulin can alter microglial behavior. Moreover, when insulin signaling is deficient in microglia, male mice are more prone to inflammatory activation of microglia, while the protective effect of estrogen in female mice partially compensates for this microglial functional impairment [15].

Microglia are also highly sensitive to iron and susceptible to ferroptosis. In the hippocampal microglia of diabetic mice, TREM1 is significantly increased, which aggravates the accumulation of Fe^2+^ in microglia and induces microglial ferroptosis via the PERK pathway of ER [100].

### 3.4. Brain Endothelial Cells

The cerebrovascular dilatory function of brain insulin primarily relies on endothelial cells. However, under BIR conditions, inflammatory factors such as TNF-α increase in BECs, triggering an inflammatory response. Gene expression analysis of isolated microvessels from T2DM and T1DM animal models revealed significant upregulation of inflammatory genes in brain tissue [101], promoting a pro-inflammatory phenotype in BECs and exacerbating the inflammatory response. Chronic hyperglycemia induced by BIR promotes the formation of advanced glycation end products (AGEs). The binding of AGEs to the receptor for AGEs (RAGE) on the surface of BECs activates the downstream NF-κB signaling pathway, driving the excessive secretion of pro-inflammatory factors and recruiting peripheral immune cells to infiltrate the brain parenchyma, thereby forming a self-amplifying inflammatory cycle [102]. Furthermore, under BIR conditions, HDAC3 becomes hyperactivated and suppresses the expression of the antioxidant gene HO-1, leading to BECs damage, the activation of inflammatory responses, and the disruption of BBB function. HDAC3 inhibitors can improve BBB permeability by activating the Nrf2 pathway [103].

BECs are tightly interconnected to form the BBB, and their dysfunction directly impacts BBB integrity, primarily through alterations in the proteins that connect BECs. On one hand, hyperglycemia and inflammatory factors (such as TNF-α and IL-1β) significantly suppress the gene expression of key junctional proteins, including Claudin-5, Occludin, and ZO-1 [104]. Transcriptomic analyses also show that gene clusters related to vascular development are markedly downregulated in BECs under BIR conditions [41]. On the other hand, stimulation by AGEs induces BECs to secrete Occludin into extracellular microvesicles, upregulates the expression of adhesion molecules such as ICAM and VCAM, increases the adhesion and migration of leukocytes across the BEC monolayer, elevates BBB permeability, and leads to the loss of structural barrier proteins [105].

Under BIR conditions, the influence of astrocytes and pericytes within the neurovascular unit on brain endothelial cells is also highly important [106]. Abnormal secretion of vascular endothelial growth factor (VEGF) by astrocytes directly acts on BECs, impairing endothelial tight junctions and increasing BBB permeability [107]; meanwhile, the loss of insulin signaling in pericytes disrupts vascular stability and angiogenesis [108]. These changes collectively exacerbate cerebral endothelial dysfunction, impair cerebrovascular regulation, and worsen BIR, forming a vicious cycle.

### 3.5. Oligodendrocytes

BIR significantly impairs the differentiation of oligodendrocyte progenitor cells (OPCs) and disrupts myelin homeostasis [109]. Under healthy conditions, insulin and IGF-1 promote the differentiation of OPCs into mature OLs via the Akt pathway, thereby driving myelination [110,111]. However, under BIR conditions, impaired insulin signaling leads to a failure in Akt pathway activation, thereby hindering cellular glucose uptake, which subsequently inhibits OPC differentiation and induces energy metabolism dysfunction in mature OLs [112]. Clinical studies have confirmed a significant reduction in the myelin water fraction (MWF) in the cerebral white matter of patients with T2DM, which shows a negative correlation with circulating insulin levels [113,114]. Kinases such as serum/glucocorticoid-regulated kinase 1 (SGK1) [115] and cyclin-dependent kinase 5 (Cdk5) [116] are overactivated under BIR conditions, triggering inflammatory responses and OL apoptosis in regions such as the hippocampus or hypothalamus, ultimately leading to myelin degradation. Administration of a Cdk5 inhibitor (e.g., TFP5) can effectively alleviate neuroinflammation and myelin degradation. Recent studies have also found that myelin injury in the hippocampus of T2DM mice precedes neuronal damage, and the former is closely associated with the cognitive deficits observed in these mice [117].

In addition to affecting OPC differentiation, BIR also indirectly disrupts myelin homeostasis through mitochondrial and endoplasmic reticulum dysfunction. Prolonged HFD-induced BIR leads to mitochondrial hyperactivation in OLs, generating excessive ROS, which results in oxidative stress and induces cellular pyroptosis. While mature OLs rely less on mitochondrial energy supply, mitochondrial damage reduces their responsiveness to brain plasticity [118], thereby hindering myelin repair. Moreover, abnormally activated ER stress interferes with the translation of myelin basic protein (MBP), directly impeding myelination.

**Figure 3:**
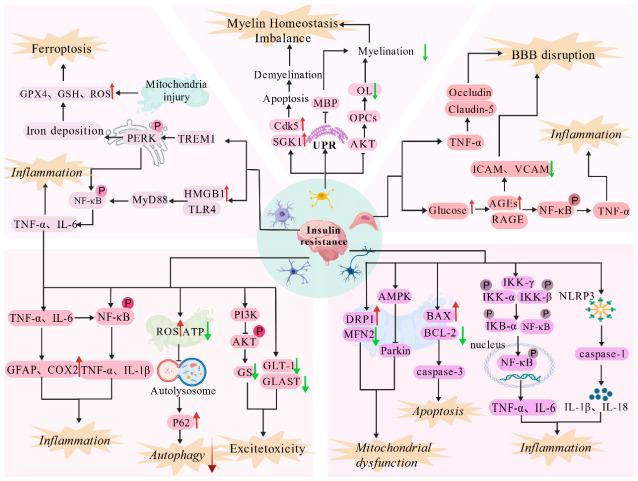
The mechanisms of BIR and neuroinflammation interaction. In neurons, BIR triggers mitochondrial fission and dysfunction, leading to reduced ATP synthesis and inducing oxidative stress. Concurrently, it activates the NF-κB pathway and the NLRP3 inflammasome, promoting the release of pro-inflammatory factors (such as TNF-α and IL-1β) and triggering apoptosis. Under BIR, astrocytes are driven toward the neurotoxic A1 phenotype, autophagy is impaired, and the downregulation of glutamate transporters GLT1/GLAST leads to excitotoxicity. Microglia are activated via pathways such as HMGB1/TLR4/NF-κB, releasing large amounts of pro-inflammatory cytokines and potentially undergoing ferroptosis. These pro-inflammatory factors can further affect astrocytes through paracrine signaling, exacerbating their neuroinflammatory responses. In BECs, hyperglycemia and AGEs–RAGE signaling activate NF-κB, suppress the expression of tight junction proteins (such as Claudin-5 and ZO-1), and compromise BBB permeability. In oligodendrocytes, impaired insulin signaling inhibits their differentiation and reduces myelination, and mitochondrial and endoplasmic reticulum dysfunction further impair their myelin repair capacity. Pro-inflammatory factors released from various cell types feed back to aggravate insulin signaling abnormalities, forming a self-amplifying vicious cycle. Created with BioGDP.com [59].

In obesity-induced diabetic models, the disruption of myelin lipid homeostasis may be more closely associated with hyperlipidemia or obesity itself rather than being directly caused by BIR [119]. Furthermore, human islet amyloid polypeptide (hIAPP) in the serum of patients with T2DM has been shown to independently induce acidosis and demyelination by impairing the function of monocarboxylate transporter 1 (MCT1) in oligodendrocytes, independent of blood glucose levels [120]. This highlights the need for more in-depth and detailed mechanistic studies on different subtypes of diabetes. The mechanisms of interaction between BIR and neuroinflammation are illustrated in Figure 3.

## 4. Therapeutic Drugs and Natural Compounds

With the continuous elucidation of the core pathogenic mechanisms of DE, targeted interventions against BIR and neuroinflammation have become the central strategy for delaying cognitive decline. In the field of drug development, conventional antidiabetic agents have demonstrated significant neuroprotective potential in both preclinical and clinical studies due to their ability to effectively modulate central insulin signaling pathways and reverse metabolic disturbances. Meanwhile, a series of novel antidiabetic drugs with multi-target effects have also provided new candidate options for the treatment of DE. In addition to conventional chemical drugs, natural compounds with broad sources and multi-pathway regulatory effects, as well as healthy lifestyle interventions focusing on diet and exercise, play a synergistic role that cannot be ignored in ameliorating DE-related cognitive impairment. However, given the heterogeneity observed in some clinical study results, it is crucial to systematically evaluate the efficacy and evidence chain of existing therapeutic approaches. To this end, this review summarizes the current major therapeutic drugs, natural compounds, and lifestyle intervention strategies for DE (Table 1), and further compiles key clinical trials—both completed and ongoing—that have investigated antidiabetic agents for the treatment of cognitive impairment in recent years (Table 2), aiming to provide a reference for future clinical translation and optimization of therapeutic strategies.

### 4.1. Therapeutic Drugs

#### 4.1.1. Biguanides

Metformin, widely used as a first-line treatment for T2DM, has demonstrated potential neuroprotective effects in previous studies. It does not increase the risk of hypoglycemia, supporting its long-term use in elderly populations. Multiple studies indicate that diabetic patients on long-term metformin therapy have a lower risk of developing dementia compared to non-users, suggesting its benefits may extend beyond glycemic control alone [121]. Furthermore, metformin use is associated with a significantly reduced incidence of delirium in elderly T2D patients, with a clear dose–response relationship observed [122]. Additionally, the Sydney Memory and Ageing Study reported that long-term metformin use in older patients can delay cognitive decline and lower the risk of dementia [123]. Animal studies further reveal that metformin significantly alleviates neuroinflammation, reduces neuronal loss in the hippocampus, and thereby improves spatial memory function [124]. These findings provide experimental support for its application in cognitive protection.

#### 4.1.2. Sulfonylurea

Sulfonylureas, as the first class of antidiabetic drugs that lower blood glucose by stimulating insulin secretion, remain widely used in clinical practice. These agents can cross the BBB and act on cerebral ATP-sensitive potassium (KATP) channels and sulfonylurea receptors, thereby modulating neuronal activity [125]. Preclinical studies have found that sulfonylureas reduce the neurotoxicity of Aβ and α-synuclein, which are key neurotoxic proteins in AD and PD, respectively, and improve learning and memory in rodent models [126]. Other research indicates that both DPP-4 inhibitors and sulfonylureas can reverse BBB leakage caused by type 2 diabetes. For example, glimepiride enhances angiogenesis and increases pericyte density in T2DM models, thereby promoting the restoration of vascular structural integrity [127], which may represent one mechanism underlying its neurorestorative effects. However, the impact of sulfonylureas on neurocognition in clinical practice remains controversial. Some studies have found that elderly diabetic patients using sulfonylureas, particularly glibenclamide, exhibit a higher risk of dementia compared to those using DPP-4 inhibitors [128].

#### 4.1.3. GLP-1 Receptor Agonists

In recent years, GLP-1 receptor agonists (GLP-1RAs) have become important therapeutic agents for T2DM. These drugs not only effectively lower blood glucose but also help manage obesity by suppressing appetite, reducing food intake, and modulating body weight. Animal studies indicate that GLP-1RAs also demonstrate neuroprotective potential, showing positive effects, particularly in improving memory function [129,130]. In analyses examining the relationship between antidiabetic drugs and cognitive impairment, multiple randomized controlled trials consistently show that GLP-1RAs can delay the decline in memory function—including in patients at the preclinical or early disease stages—and reduce their future risk of dementia [131,132]. Furthermore, new-generation dual or triple receptor agonists can simultaneously target GLP-1 receptors (GLP-1R), glucose-dependent insulinotropic polypeptide receptors (GIPR), and glucagon receptors (GCGR). By synergistically regulating energy expenditure and glucose homeostasis, they may offer more comprehensive metabolic and neuroprotective benefits. For instance, mazdutide, a dual GLP-1/GCGR agonist, has been shown to improve cognitive function through multiple mechanisms in models of obesity and type 2 diabetes [133]. These findings suggest that GLP-1RAs and related multi-target drugs may positively influence cognitive impairment, potentially through mechanisms such as reducing neuroinflammation, highlighting their promising value in the prevention and treatment of diabetic encephalopathy.

#### 4.1.4. Sodium-Glucose Cotransporter-2 Inhibitors

Sodium-glucose cotransporter-2 inhibitors (SGLT2Is) represent a novel class of hypoglycemic agents that improve systemic metabolic status by inhibiting renal glucose reabsorption and promoting urinary glucose excretion. A growing body of evidence suggests their potential neuroprotective role in various neurodegenerative diseases and associated cognitive decline. Recent studies indicate that dapagliflozin counteracts ROS-dependent neuronal apoptosis, upregulates the GDNF and its downstream PI3K/AKT/GSK-3β (Ser9) pathway, and suppresses neuroinflammation by inhibiting the activation of the NF-κB pathway and TNF-α levels [134]. Furthermore, SGLT2Is alleviate cerebral oxidative stress, reduce Aβ deposition and tau hyperphosphorylation, improve brain energy metabolism and mitochondrial function, and enhance neuronal plasticity [135]. A large cohort study involving T2DM patients aged 40–69 years demonstrated that the use of SGLT2 inhibitors, compared to DPP-4 inhibitors, is associated with a reduced risk of cognitive impairment [136], with the effect being more pronounced in older populations. Although SGLT2Is hold theoretical potential for synergistically regulating glucose homeostasis and neuroinflammation, further exploration of their multi-target therapeutic strategies for DE is warranted.

#### 4.1.5. Thiazolidinedione

Thiazolidinediones (TZDs), as peroxisome proliferator-activated receptor γ (PPARγ) agonists, have demonstrated potential value in the treatment of DE in recent years. Their mechanisms of action primarily involve improving insulin resistance, alleviating neuroinflammation and oxidative stress, thereby protecting central nervous system function. For example, pioglitazone can exert neuroprotective effects in diabetic rats by modulating cerebral SIRT-1 signaling, improving oxidative stress, and targeting altered mitochondrial biogenesis by balancing pro-apoptotic and anti-apoptotic mediators [137]. Preclinical studies further confirm that pioglitazone alleviates diabetes-related cognitive dysfunction by enhancing cognitive performance and reducing blood glucose levels, neuroinflammation, and oxidative stress in diabetic rats [138]. Future research should focus on developing centrally specific PPARγ agonists or combination therapies (such as with GLP-1 receptor agonists) to provide new targeted treatment strategies for DE.

#### 4.1.6. Intranasal Insulin

Multiple studies have demonstrated that intranasally administered insulin bypasses the BBB, entering brain tissue directly via the olfactory or trigeminal pathways and achieving rapid and widespread distribution within the brain. It has shown neuroprotective effects in both preclinical research and clinical trials [139,140]. Compared to non-diabetic individuals, patients with T2DM receiving intranasal insulin treatment showed overall greater improvement in walking speed, executive function, and verbal memory [141]. A double-blind, randomized controlled trial revealed that compared to the placebo group, intranasal administration of regular insulin significantly improved patients’ memory after two and four months of treatment and reduced the tau-p181/Aβ42 ratio in the brain [142]. However, there remains some controversy regarding the efficacy of intranasal insulin for cognitive impairment in DE. A meta-analysis indicated that while intranasal insulin showed positive trends in some aspects—such as improved verbal memory in APOE4-negative Alzheimer’s disease patients—its overall effect on cognitive impairment did not reach statistical significance [143]. Given the potential of intranasal insulin, future research should involve larger dose studies with careful selection of insulin types and patient populations.

### 4.2. Natural Compounds

Medicinal plants, as natural products, demonstrate broad application prospects in the prevention and treatment of diabetes and its complications. Their therapeutic value is primarily attributed to their bioactive constituents. Investigating the mechanisms of action of these active components may provide new insights for the prevention and management of DE.

#### 4.2.1. Curcumin

Curcumin is a natural polyphenolic compound extracted from the rhizomes of the turmeric plant (*Curcuma longa*). Its clinical application has long been limited by low bioavailability and poor ability to cross the BBB [144]. Consequently, research on curcumin for the treatment of DE currently remains primarily at the preclinical stage using animal models. Multiple studies have shown that oral administration of curcumin (common doses ranging from 50 to 200 mg/kg/day) can significantly improve cognitive dysfunction in diabetic animal models. Its mechanisms include inhibiting glial cell activation and the production of inflammatory factors, repairing hippocampal neuronal structure and synaptic plasticity, activating the Nrf2-ARE pathway, alleviating endoplasmic reticulum stress, and modulating blood glucose and lipid levels [145,146,147]. Research has also found that in diabetic rat models, curcumin and its analog A13 improve cerebral inflammation and oxidative stress by modulating the classical NF-κB/p65 pathway [148]. In summary, curcumin can serve as a potential neuroprotective agent for DE, helping to prevent diabetes-induced pathological damage.

#### 4.2.2. Resveratrol

Resveratrol is a non-flavonoid polyphenolic compound found in red wine, grapes, nuts, and berries. It is well established that resveratrol exerts protective effects against diabetes-induced cognitive dysfunction. Multiple studies have demonstrated that resveratrol can improve mitochondrial function in the neurons of diabetic mice, alleviate neuroinflammation by modulating microglial activation and cytokine release, reduce oxidative damage by scavenging ROS, and may exert antioxidant and anti-inflammatory effects via insulin signaling pathways [149,150,151,152,153]. In an STZ-induced in vitro neurodegeneration model, resveratrol concurrently activates the SIRT1-PGC-1α axis to promote mitochondrial biogenesis, inhibits excessive autophagy, and restores the PI3K/Akt insulin signaling pathway, thereby reversing neuronal insulin resistance and cellular damage [154]. Furthermore, resveratrol can reverse oleic acid-induced glucolipid metabolic disorders and cellular injury by activating the Wnt/β-catenin signaling pathway [155]. Recent advances in targeted therapies for BIR include a study utilizing brain-targeting peptide-modified chitosan nanoparticles to deliver resveratrol. This approach demonstrated that resveratrol can simultaneously suppress cerebral inflammation, enhance insulin signaling, and remodel the gut microbiota in a mouse model of obesity-related Alzheimer’s disease, thereby improving BIR [156].

#### 4.2.3. Berberine

Berberine is an isoquoline alkaloid extracted from plants such as *Coptis chinensis* and *Phellodendron amurense*. While clinically used for treating intestinal infections and diarrhea, its potential in preventing and managing DE has garnered increasing attention as research into the pathogenesis of DE deepens. Berberine has been shown to improve cognitive dysfunction and neuropathological damage in DE, which is attributed to its ability to activate the PI3K/Akt and MAPK signaling pathways, thereby ameliorating BIR and neuronal function [157,158]. Recent research has, for the first time, revealed that berberine can inhibit the production of δ-valerobetaine (δ-VB) by gut microbiota, interrupt the TLR-4/MyD88/NF-κB inflammatory pathway in vascular endothelial cells, and restore impaired BBB and gut barrier function in DE mice [159], thereby alleviating neuroinflammation and oxidative stress. Current studies are employing cocrystal technology to overcome the limitation of berberine’s low bioavailability [160]. With the development of novel drug delivery systems and the conducting of larger-scale clinical trials, berberine is expected to become an important complementary agent in the comprehensive treatment of DE.

### 4.3. Lifestyle Interventions

In recent years, lifestyle interventions have gained increasing prominence as a non-pharmacological strategy for the prevention and management of DE. A study based on the UK Biobank revealed that a healthy lifestyle can significantly attenuate diabetes-associated accelerated brain aging, with non-smoking, moderate alcohol consumption, and high levels of physical activity serving as key protective factors [161].

Dietary control, a cornerstone of diabetes management, also plays a crucial role in influencing diabetes-related cognitive impairment. Substantial literature confirms that a HFD increases BBB permeability, disrupts tight junction integrity, and induces neuroinflammation-related hippocampal dysfunction. Notably, the use of FFAR3 agonists or vitamin D supplementation has been shown to mitigate HFD-induced BBB damage and improve cognitive impairment [162,163,164]. Furthermore, intermittent fasting has been found to suppress HFD-induced BBB disruption in the hippocampus of mice and reduce microglial GAL3 and astrocytic LCN2 protein levels, thereby inhibiting neuroinflammation and ameliorating cognitive deficits [165]. Additionally, prebiotics, probiotics, or synbiotics can improve hippocampal plasticity via the gut–brain axis, alleviate cerebral mitochondrial dysfunction, and reduce microglial activation in HFD-fed rats, ultimately restoring cognitive function [166].

It is well established that structured and scientifically designed exercise can significantly promote physical health and reduce the risk of exercise-related injuries. Aerobic exercise, such as brisk walking for 40 min three times per week, has been shown to significantly improve non-verbal memory, verbal memory, and attention in patients with T2DM. Compared to sedentary db/db mice, aerobic exercise improves cognitive impairment by activating the AMPK/SIRT1 signaling pathway and inhibiting the JAK2/STAT3 pathway [167]. Furthermore, studies have found that aerobic exercise modulates the gut microbiota via the GBA, thereby helping to alleviate cognitive dysfunction in T2DM mice [168]. In an HFD-induced mouse model of cognitive decline, swimming intervention was found to reduce inflammatory factors, inhibit the JNK/IRS-1/PI3K/Akt signaling pathway, and activate the PGC-1α/BDNF pathway, thereby suppressing neuronal apoptosis and ameliorating cognitive decline in the mice [169]. Current exercise recommendations predominantly advocate a combined model integrating aerobic exercise with resistance training. Recent research has found that combined aerobic and resistance training is effective in reducing BMI, improving glycemic control, and decreasing inflammatory markers through mechanisms involving the AMPK and mTOR pathways.

In summary, the prevention and treatment of DE have evolved into a comprehensive strategy integrating “multi-target drug intervention” and “non-pharmacological lifestyle modulation.” In terms of pharmacotherapy, agents such as metformin, GLP-1Ras, and SGLT2Is are primarily used to maintain peripheral metabolic homeostasis. These are combined with PPARγ agonists, intranasal insulin, and natural products like berberine, collectively exerting central sensitizing, anti-inflammatory, antioxidant, and BBB-repairing effects. In the realm of non-pharmacological interventions, the importance of scientific dietary and exercise regimens has become prominent. Intermittent fasting, probiotic supplementation, and vitamin D administration can protect the BBB by modulating the GBA and suppressing neuroinflammation. Meanwhile, combined aerobic and resistance training effectively reverses cognitive decline by activating pathways such as AMPK and BDNF. The clinical translation of therapeutic agents for DE faces several limitations, including poor central bioavailability, substantial differences between animal models and human pathology, a lack of specific biomarkers, and poor long-term adherence to combination therapies. In the future, clinical practice should prioritize drugs that possess both glucose-lowering and brain-protective effects while avoiding agents that increase cognitive risk. Research efforts should focus on central-specific agonists, multi-target combination strategies, and the use of brain-targeted nanodelivery and cocrystal technologies to overcome the bioavailability bottlenecks of natural products, thereby providing more precise preventive and therapeutic options for DE.

**Table 1:** Summary of potential therapeutic drugs for diabetic encephalopathy.

Classification	Drug	Model	Mechanism	Influence	References
Synthetic drug	Biguanides	T2DM/Animal model	Activate the AMPK pathway to alter mitochondrial metabolism	Restore glucose homeostasis, delay cognitive decline, reduce neuroinflammation, and improve spatial memory	[123]
	Sulfonylureas	T2DM	Modulate neuronal KATP channels; reduce the neurotoxicity of Aβ and α-synuclein	Enhance learning and memory abilities	[126]
	GLP-1 receptor agonist	Diabetic patients/animal models	Activate GLP-1 receptors to enhance insulin secretion	Delaying memory decline	[130,131]
	SGLT2Is	T2DM/Rat Models of Diabetic Neuropathy	Regulate the IGF-1R signaling pathway, inhibit the NF-κB pathway, and alleviate neuroinflammation and oxidative stress	Reducing the risk of cognitive impairment	[134]
	Thiazolidinediones	Diabetic rat model	Activate PPARγ to improve central insulin resistance; regulate brain SIRT-1 signaling to mitigate oxidative stress. SIRT-1	Lower blood sugar, enhance cognitive function	[137]
	Intranasal insulin	T2DM/AD	Deliver insulin directly to the brain to improve central insulin resistance	Improve cognitive function	[142]
Active ingredients of Chinese medicine	Curcumin	Diabetic rat/mouse model	Downregulate the NF-κB/p65 pathway; activate the Nrf2-ARE pathway	Improve pathological damage and inflammation in brain tissue	[148]
	Resveratrol	Diabetic mouse model	Activate the SIRT1-PGC-1α axis and Wnt/β-catenin signaling pathway to reduce neuronal apoptosis	Protect against diabetes-induced cognitive impairment and improve spatial learning and memory abilities	[154]
	Berberine	DE mouse model	Activate PI3K/Akt and MAPK signaling pathways; downregulate TLR-4/MyD88/NF-κB	Improve brain-blood barrier integrity and cognitive function in model mice, protect the blood–brain barrier, and repair the cerebral vascular network	[159]

**Table 2:** Selected clinical trials of antidiabetic drugs for cognitive impairment.

Drug Classification	Drug Name	Phase	Status	Study Population	Key Outcomes	Study Title	Registration Number	Website URL
Biguanides	Metformin	Phase III	Completed	Individuals at risk of cognitive decline or exhibiting early memory impairment	Primary focus on long-term prevention of cognitive decline, assessing domain-specific cognitive scores (particularly memory retention), and tracking neuro-progression.	Preventing Cognitive Decline with Metformin: the MetMemory Study	NCT04511416	https://clinicaltrials.gov/study/NCT04511416 (accessed on 9 June 2026)
GLP-1Ras	Liraglutid	NA	Completed	Overweight/Obese T2DM patients inadequately controlled with metformin monotherapy	Compares the distinct impacts of three drug classes on cognitive scores, olfactory function, and localized odor-induced neuro-activation via task-based fMRI scans.	Effects of Liraglutid, Dapagliflozin and Acarbose on the Cognitive Function, Olfactory Function, and Odor-induced Brain Activation in Overweight/Obese T2DM Patients Controlled Inadequately with Metformin Monotherapy.	NCT03961659	https://clinicaltrials.gov/study/NCT03961659 (accessed on 9 June 2026)
Glucagon (GCG)/glucagon-like peptide-1 (GLP-1) dual receptor agonists	Mazdutide	Phase III	Ongoing	T2DM patients with concurrent early-stage dementia (MCI or mild AD)	To investigate the efficacy of dual-receptor synergy on slowing cognitive deterioration and reversing central energy failure.	GLP-1/GCG Dual Agonist in Type 2 Diabetes with Early Dementia (LIGHT-COG Study)	NCT07083154	https://clinicaltrials.gov/study/NCT07083154 (accessed on 9 June 2026)
Sodium-Glucose Cotransporter 2 Inhibitors	Henagliflozin	NA	Completed	Patients with T2DM and mild cognitive impairment (MCI)	Evaluates improvements in cognitive scores (MoCA/MMSE) and monitors changes in cerebral blood flow and functional connectivity via multimodal brain MRI.	Effects of Henagliflozin on the Brain Function in T2DM Patients with Mild Cognitive Impairment: a Randomized, Parallel Controlled Clinical Trial	NCT06085703	https://clinicaltrials.gov/study/NCT06085703 (accessed on 9 June 2026)
Thiazolidinedione	Pioglitazone	Phase IV	Completed	Schizophrenia patients suffering from comorbid antipsychotic-induced metabolic syndrome	Evaluates dual efficacy in reversing systemic glucolipotoxicity (lipid profiles/HOMA-IR) and enhancing psychiatric cognitive domains (working memory, executive function) via central metabolic correction.	Pioglitazone as a Treatment for Lipid and Glucose Abnormalities In Patients with Schizophrenia	NCT00231894	https://clinicaltrials.gov/study/NCT00231894 (accessed on 9 June 2026)
Dipeptidyl Peptidase-4 Inhibitors	Sitagliptin	NA	Completed	Middle-aged and older T2DM patients at elevated risk of cognitive regression	Monitors cognitive trajectory changes (MoCA/MMSE scores), and assesses macro-vascular brain preservation via neuroimaging markers.	Investigating the Protective Effect of Newer Antidiabetic Drugs on Cognitive Decline in Diabetic Patients	NCT05347459	https://clinicaltrials.gov/study/NCT05347459 (accessed on 9 June 2026)
Dipeptidyl Peptidase-4 Inhibitors	Vildagliptin	Phase IV	Completed	Older adults (geriatric population) with T2DM and concurrent MCI	Assesses stabilization or improvement in global cognitive performance (MoCA/MMSE) while evaluating safety and the prevention of hypoglycemia-induced cognitive worsening.	Vildagliptin in Older Adults with Diabetes and Mild Cognitive Impairment	NCT03819127	https://clinicaltrials.gov/study/NCT03819127 (accessed on 9 June 2026)
Insulin	Intranasal Insulin	Phase II	Completed	Adults with Amnestic MCI or mild-to-moderate Alzheimer’s disease	Demonstrated preservation of memory (delayed story recall), improved regional cerebral glucose metabolism (via PET scans), and stabilization of CSF biomarkers.	SNIFF 120: Study of Nasal Insulin to Fight Forgetfulness (120 Days) (SNIFF 120)	NCT00438568	https://clinicaltrials.gov/study/NCT00438568 (accessed on 9 June 2026)

## 5. Conclusions

As a severe chronic central complication of T2DM, DE poses a major challenge to global healthcare systems and socioeconomic development. This review systematically elucidates the central role of BIR and neuroinflammation in the pathogenesis and progression of DE, as well as their complex interplay. Distinct from previous reviews that focused on a single mechanism or a single drug, this review, from the perspective of “repurposing antidiabetic drugs,” integrates preclinical and clinical trial evidence, clearly proposing that BIR and neuroinflammation can serve as dual therapeutic targets for DE. It also systematically summarizes relevant studies from various clinical trial registration platforms, thereby providing a theoretical basis for the development of future multi-target therapeutic strategies.

However, BIR is not an isolated pathological event. First, most mechanistic studies rely on animal models or in vitro experiments, whose translational value to human DE is limited. Second, the precise causal relationship and regulatory network between BIR and neuroinflammation have not yet been fully elucidated. Furthermore, the impact of sex-, age-, and intergenerational-related factors on DE susceptibility and treatment response remains systematically understudied. These issues collectively represent challenges that urgently need to be addressed in future research.

To overcome the aforementioned limitations and achieve more precise prevention and treatment, future cutting-edge research urgently needs to expand and deeply explore several emerging directions. First, there are significant sex differences in the process by which metabolic disorders lead to cognitive decline, and the interplay between these sex differences and neuroendocrine regulation cannot be ignored. Future studies should thoroughly investigate sex-based differences in susceptibility to BIR, microglial activation phenotypes, and maintenance of BBB integrity, with particular emphasis on elucidating the interaction network between peripheral sex hormones such as estrogen and androgen and central signaling pathways including PI3K/Akt and MAPK, thereby establishing personalized, sex-specific precision prevention and treatment strategies for DE. This peripheral-to-central regulatory mechanism is particularly evident in the gut–brain axis (GBA). From the perspective of peripheral–central crosstalk, a core future task is to elucidate how gut microbiota dysregulation remotely induces cerebral neuroinflammation—via the vagus nerve, the circulatory system, or metabolites (e.g., short-chain fatty acids, bile acids) that cross the BBB. Unraveling this mechanism will provide novel targets for treating central disorders through gut-based interventions. However, regardless of which peripheral or central targets are identified, how to efficiently and safely deliver drugs across the BBB remains an engineering bottleneck for the neuroprotective effects of metabolic drugs. Therefore, the development of advanced brain-targeted drug delivery systems and specific administration routes has become a top priority for future research. Future research should focus on developing novel brain-targeted biological nanoparticle delivery systems that exploit receptor-mediated transcytosis to achieve precise central delivery of peripherally administered drugs. Concurrently, technological upgrades to specific delivery devices such as nasal sprays should be prioritized, leveraging the olfactory and trigeminal nerve pathways to enable direct drug delivery to the central nervous system, which holds irreplaceable clinical value for directly repairing impaired central signal transduction and rescuing synaptic damage. Given the complex multifactorial pathology of DE, the integration of such engineering breakthroughs with modern multi-target synergies and the deep convergence of Chinese and Western medicine may prove more effective. On one hand, the combined application and multi-target synergy of modern small-molecule drugs—such as the co-administration of GLP-1 analogs and SGLT2 inhibitors, or the clinical translation of novel dual GLP-1/GCG receptor agonists—should be accelerated. On the other hand, the holistic advantages of traditional Chinese medicine (TCM) formulas, characterized by their multi-component, multi-target, and synergistic microenvironment-remodeling properties, should be fully utilized.

In summary, the onset and progression of DE represent a complex pathological process involving multiple intertwined factors, with the vicious cycle between BIR and neuroinflammation playing a central bridging role. Future research urgently needs to overcome existing mechanistic heterogeneity and clinical translation bottlenecks by integrating cutting-edge perspectives such as sex differences and the gut–brain axis (GBA), along with strategies combining Chinese and Western medicine, multi-target synergies, and advanced brain-targeted drug delivery engineering, to achieve precise and efficient prevention and treatment of DE. This will not only break down traditional multidisciplinary barriers but also provide a broad and highly translationally valuable scientific foresight for the development of novel clinical drugs and the formulation of comprehensive treatment strategies.

## Data Availability

No datasets were generated or analyzed during the current study.
